# Knowledge of the Patients’ Bill of Rights and Influencing Factors Among University Nursing Students

**DOI:** 10.7759/cureus.38433

**Published:** 2023-05-02

**Authors:** Sharifa Al Syed, Ebtsam A Abou Hashish, Eman Bajamal, Lamees Abdaljabbar, Nouf Alammari, Rehab Alotaibi, Shaima Alfaifi, Hatun Alrudayni

**Affiliations:** 1 College ‎of Nursing - Jeddah, King Saud bin Abdul-Aziz University for Health Sciences, King Abdullah International Medical Research Center, Jeddah, SAU; 2 Faculty of Nursing, Alexandria University, Alexandria, EGY

**Keywords:** saudi arabia, nursing, nursing students, ethics, patients’ bill of rights

## Abstract

Background

Nursing students, the future nursing workforce, are expected to be exposed to ethically challenging situations in their workplaces, and they must be knowledgeable about patients’ rights to provide holistic care. However, limited research was cited on the knowledge of nursing students regarding the Bill of Rights and the factors influencing their knowledge.

Purpose

This study aimed to assess nursing students’ knowledge of the patients’ bill of rights and determine what factors influence this knowledge in Saudi Arabia.

Methods

A descriptive cross-sectional research study was conducted with a convenience sample of nursing students (N = 210) in a Saudi nursing college. Data were collected using a questionnaire that consists of three parts: demographic characteristics, knowledge of the patients’ bill of rights, and six open-ended and reflective questions. Descriptive statistics and response analysis are used.

Results

The statistics showed that the total knowledge score about patients’ rights ranged from 19 to 34, with a mean of 26.11 ± 2.32, among nursing students. About two-thirds of students reported adequate knowledge (n = 118, 65.5%), compared to those who had inadequate knowledge (n = 62, 34.5%). In addition to academic level and age, students reflected on many factors that shape their ethical knowledge, such as integrated, interprofessional learning experiences, workshops, a supportive and ethical learning environment, and the presence of an ethical committee.

Conclusion

Continuous efforts to foster ethics education with inspiring learning content and innovative instructional material are vital to improving nursing students’ knowledge and readiness. Interprofessional education (IPE) sessions and awareness programs are effective strategies to improve their ethical awareness and knowledge.

## Introduction

The healthcare system aims to provide high-quality care to all patients while respecting their rights [1]. Given the patient’s unique circumstances, which easily expose him to violations and vulnerability, the healthcare system must emphasize human rights, particularly the patient’s dignity as a human being [2]. Thus, the patients’ bill of rights is the most pressing ethical issue for healthcare professionals [1]. The Bill of Rights is a set of claims that determine the status and needs of patients during the provision of health services and the obligations of the healthcare providers toward the patients as well as their relatives [3]. Nurses and students as a future workforce should be aware and knowledgeable about the Bill of Rights to provide quality patient care [1].

The concept of patients’ rights was developed based on the Universal Declaration of Human Rights adopted by the United Nations General Assembly in 1948, which explicitly states that each human being on earth has an inherent right to own his or her life, freedom, privacy, free development, and respect for dignity within society [3]. The main objective is to safeguard the patient’s autonomy from any interference by other individuals [4]. Hence, the patients’ bill of rights is considered a guide to choosing and ensuring the best future decisions that attain the patients’ benefits and can be the starting point for comprehensive interest in patients’ rights and a correct definition of the relationship between healthcare receivers and providers [5]. Patients’ rights may vary by country, authority, and cultural and social norms [6], but they guarantee proper services, patient independence and decision-making rights, privacy, and access to a complaint system [7]. It also improves patient satisfaction; reduces treatment costs, hospital stays, and irreversible physical or emotional harm; and allows patients to participate in the treatment plan [5].

With a close look at the Islamic perspective, the Holy Quran and Sunnah, the fundamental resources for all Muslims, reflect the importance of human rights in Islam. From the moment of birth, all human beings are free, have similar rights, and are equally graceful [8]. The Ministry of Health (MOH) published a new edition of the Saudi Patients’ Bill of Rights (PBR) on December 27, 2011 [9]. According to the Patients’ Bill of Rights (PBR), patients in Saudi Arabia are guaranteed access to quality care that is tailored to their specific needs, as well as the right to be informed about and participate in their care, lodge complaints, and have their privacy protected. The bill mandates that patients be given the option to change or refuse their treatments and that they be made aware of any potential risks. The bill also requires that patients receiving care in the private sector be informed of their treatment’s total cost in advance and that they not be subjected to any medical management without their informed consent [9,10].

Nursing and patient rights

Modern technological, medical, and nursing advancements have created several moral, professional, and ethical dilemmas that nurses must resolve based on their own ethical knowledge and values while maintaining and advocating for patients’ rights [11]. Also, nursing students are expected to frequently run into unanticipated ethical dilemmas during their learning experiences and clinical training with patients and staff. To handle these situations effectively, they must be educated and prepared professionally [11,12]. Their lack of awareness of patients’ rights might lead to their inability to recognize patients’ legal and ethical issues and reduce the quality of the services they provide when they practice their future roles [2]. Therefore, nursing students should be trained and prepared to handle unexpected ethical dilemmas during clinical training with patients and staff. Preparing nursing students for ethical issues in their careers has been shown to be important [13]. However, limited research was cited on the knowledge of nursing students regarding the Bill of Rights and factors influencing their knowledge while they were still studying, especially among Saudi students. Our study aims to fill this research gap and provide information about the basic knowledge base of patients’ bill of rights and how to improve it among nursing students.

An efficient healthcare system is one that involves active interactions and satisfactory relationships between nurses and patients based on the patients’ rights [3]. Having this importance, previous research concluded that nursing curricula should give adequate concern to the patients’ rights as a framework of ethical care [11,12]. Education and training can influence nursing students’ clinical behavior by increasing ethical and moral awareness. According to Abou Hashish and Ali Awad [14], nursing educators help students and nurses learn about the code of ethics, professional values, and patients’ rights and encourage them to think about ethical dilemmas and apply ethical reflection. Because patients’ rights advocacy responsibilities are the cornerstone of nursing ethics, the goal of nursing students’ education is to not only provide them with knowledge about patients’ rights but also to enable them to engage in patient advocacy. It is crucial that prospective nurses receive ethics training to prepare them for this position [11,12,14].

Significance of the study

To make sure the rights of patients are protected, more attention is required to assessing the knowledge of health providers, including the current and prospective nursing workforce [15]. Internationally, knowledge of the patient’s bill of rights has gained great importance but has only been represented in a few research studies. For instance, in Egypt, El-Shimy et al. [16] investigated the perception of patients in comparison with nurses regarding patients’ bills of rights, and Kupcewicz et al. [3] included nursing students from Poland, Spain, and Slovakia, in addition to Kiong et al. [4] in Malaysia and Dehghan et al. [5] in Iran. Likewise, Vivian et al. [17] measured the awareness of medical students of the patients’ bill of rights. It is worth mentioning that these studies emphasize that the concept of patients’ rights should be included in undergraduate and postgraduate education.

Despite the fact that patients’ bills of rights are critical to an efficient healthcare system and quality nursing care, little research has been conducted in Saudi Arabia to assess nursing students’ knowledge of patients’ bills, and few researchers have assessed it among patients [18-21] and doctors [10] only, with no focus on nurses or nursing students. Instead, research gave interest in physicians’ knowledge about patients’ bills of rights, as if indicating that only doctors should know this and advocate for these rights. Up to the current researchers’ knowledge, there is only one study that included medical students in Saudi Arabia, by Al Anazi et al. [22]. Believing in the fact that healthcare services are not introduced only by doctors in Saudi Arabia and nurses represent a number that surpasses that of doctors, this creates a great research gap that should be filled [3]. To the knowledge of the researchers, there is no study that assesses the level of knowledge about patients’ bill of rights among nursing students in Saudi Arabia. Therefore, this study aims to assess the level of knowledge about the patients’ bill of rights and influencing factors among nursing students at the College of Nursing, King Saud bin Abdul-Aziz University, Jeddah, Saudi Arabia. This study aimed to fill this gap by providing essential data to guide nurse educators in improving nursing ethics education strategies for undergraduate nursing students.

Aim of the study

This study aimed to assess nursing students’ knowledge of the patients’ bill of rights and determine what factors influence this knowledge in Saudi Arabia.

## Materials and methods

Design and setting

The study utilized a descriptive cross-sectional design and was conducted at the College ‎of Nursing - Jeddah (CON-J), affiliated with King Saud bin Abdul-Aziz University for Health Sciences, National ‎Guard Health Affairs, Jeddah, Saudi Arabia.

Participants

The researchers invited all undergraduate nursing students who were in their third and fourth years (N = 295) during the academic year 2022-2023. They represented the eighth, ninth, 10th, 11th, and 12th academic levels. The inclusion criteria were female students enrolled in the College of Nursing - Jeddah, students who studied the course of ethics, and students who voluntarily participated in the study. Students who had not taken an ethics education course yet were excluded. The sample size was estimated using G*Power software, which allows sample size analysis and high-precision power and computes the power values for sample size (80%), effect size, and alpha levels (0.05); the required suggested sample size was 168. Out of the 295 students, 15 were invited to the pretesting of the study instruments. To get the required sample, we invited all eligible students, and out of the 280 students, 180 returned a complete study questionnaire, which exceeded the required sample size.

Study instrument

A three-part self-administered questionnaire was used to collect data for this study.

Part 1: Sociodemographic Data

This part included two questions asking about the students’ age and academic level.

Part 2: Patients’ Bill of Rights (PBR)

This part was designed based on the patients’ bill of rights document that was published in 2011 by the Ministry of Health (MOH) in the Kingdom of Saudi Arabia (KSA) [9]. It comprises 34 statements to explore the participants’ knowledge regarding PBR. Responses to these statements are scored as “agree,” “disagree,” or “don’t know.” The correct answer is given 1 as a score, and the incorrect response or “don’t know” response is given zero as a score. The total score was computed for each participant. The level of knowledge was categorized as adequate if the score was ≥75 (score of 26-34) and inadequate knowledge if the score was <75 (score of 25).

Part 3: Reflective Experience

This section included six open-ended questions asking about different factors that shape students’ ethical knowledge and awareness of the Bill of Rights: (1) what facilitating factors influence nursing students’ knowledge of patients’ bills of rights; (2) what are the barriers or negative factors that influence nursing students’ knowledge of patients’ rights; (3) could you reflect on a situation or a story that shaped your perception of the importance of the patients’ bill of rights; (4) how do you rate the importance of knowing the patients’ bill of rights during your academic studies, from 1 to 5 (from very important to not important), and why; (5) to what extent do you agree that ethics education is important in undergraduate education, from 1 to 5 (from strongly agree to strongly disagree), and why; and (6) which learning experience affected your knowledge more, and why?

Responses on this part were summarized using frequency and percentage and represented by students’ sentences and words.

Validity and reliability

The questionnaire used in this study was used previously in Saudi studies and was indicated to be valid and reliable in the Saudi context. Two consultants in medical ethics confirmed its validity [10,22]. However, we tested it for content validity by five academic experts who proved it a valid tool. Also, the tool’s reliability was tested using Cronbach’s alpha to calculate the overall internal consistency for the 34-item scale, and the coefficient was 0.78. Additionally, the pretesting of the tool yielded no modifications to the final instrument.

Data collection

After receiving Institutional Review Board (IRB) approval from King Abdullah International Medical Research Center (KAIMRC), the researchers distributed the questionnaire to nursing students who agreed to participate in the study. The researchers clarified the study’s purpose to all participants. The time to fill out the complete question was estimated to be 30 minutes. Data were collected for two months during the academic year 2022-2023.

Ethical considerations

The study obtained KAIMRC IRB approval (SP22J/154/12). The researchers clarified the study’s purpose and the participants’ right to refuse or withdraw at any time without affecting their classes or grades. The researchers obtained participants’ informed consent to participate in the study and assured data privacy and confidentiality. The participants’ anonymity was also granted.

Data analysis

Data were coded by the researchers and statistically analyzed using Statistical Package for the Social Sciences (SPSS) version 25 (IBM SPSS Statistics, Armonk, NY, USA). Cronbach’s alpha correlation coefficient was used to test the study’s tools for internal reliability. The researchers used frequencies and percentages to describe the demographic characteristics, descriptive statistics (means and standard deviation (SD)) to summarize the results, and Pearson’s correlation to assess the relationship between studied variables. Summative scores are converted into percentages. Responses to the open-ended questions were also summarized and arranged in descending order by frequency and percentages. The statistical significance level was set at p ≤ 0.05.

## Results

Nursing students’ age and academic level

A total of 180 nursing students were included in the final analysis for this study. All were female Saudi students. Participants’ age ranged from 19 to 26 years with a mean of 21.44 ± 1.58 years with the highest percentage (47.8%) belonging to the age group 21-22 years. Students represented the eighth, ninth, 10th, 11th, and 12th academic levels at 17.2%, 17.8%, 14.4%, 18.9%, and 31.72%, respectively (Table 1).

**Table 1:** Nursing students’ distribution per age and academic level (N = 180)

Demographic variable	Number	%
Age		
19-20	52	28.9
21-22	86	47.8
23-24	37	20.5
25-26	5	2.80
Academic level		
8^th^ level	31	17.2
9^th^ level	32	17.8
10^th^ level	26	14.4
11^th^ level	34	18.9
12^th^ level	57	31.7

Perceived knowledge about patients’ bill of rights

Table 2 and Table 3 illustrate the knowledge level and scores about patients’ bill of rights. The total knowledge score about patients’ bill of rights ranged from 19 to 34 with a mean of 26.11 ± 2.32. In addition, about two-thirds of the students have adequate knowledge (n = 118, 65.5%), compared with students who have inadequate knowledge (n = 62, 34.5%). In addition, out of the 34 items, 25 were reported by students with adequate knowledge level (more than 75%) where the items scores ranged between 76.1% and 99.4% compared to nine items that had a knowledge level of less than 75%.

**Table 2:** Overall level of knowledge of patients’ bill of rights among nursing students (N = 180) Adequate knowledge = score ≥ 75 (score of 26-34) Inadequate knowledge = score < 75 (score of 25-0)

Level of knowledge	Number	%
<75 (inadequate knowledge)	62	34.5
≥75 (adequate knowledge)	118	65.5

**Table 3:** Knowledge mean score and percentage of patients’ bill of rights among nursing students (N = 180)

Rights	Mean	Standard deviation	Agree (number (%))	Disagree/I don’t know (number (%))
1. Patients are not required to be treated with courtesy and respect during times of emergency.	0.48	0.50	86 (47.8)	94 (52.2)
2. The patient should know the identity and professional status of the healthcare providers responsible for his treatment.	0.92	0.27	165 (91.7)	15 (8.3)
3. A patient is entitled to know the name of the physician performing the procedure except in an emergency case.	0.91	0.29	163 (90.6)	17 (9.4)
4. Patients are entitled to know a method of contacting their treating physician.	0.96	0.19	173 (96.1)	7 (3.9)
5. The patient should be notified about the diagnosis and all treatment updates in understandable language.	0.99	0.07	179 (99.4)	1 (.6)
6. Patient’s culture and beliefs should be respected even if it was against medical advice.	0.96	0.20	172 (95.6)	8 (4.4)
7. A patient may have the possibility of obtaining a second opinion within the same hospital or another.	0.96	0.19	173 (96.1)	7 (3.9)
8. Patients should be examined in a private examination room.	0.92	0.27	165 (91.7)	15 (8.3)
9. When examining a patient, a third party should be present.	0.82	0.38	147 (81.7)	33 (18.3)
10. Treatment options should be discussed within the health team; patients are only entitled to know the treatment plan.	0.72	0.45	129 (71.7)	51 (28.3)
11. The patient’s medical record can be accessed by the healthcare team - researchers and other hospital staff.	0.51	0.50	91 (50.6)	89 (49.4)
12. A doctor can disclose an adult patient’s information to anyone upon his permission.	0.82	0.38	148 (82.2)	32 (17.8)
13. A doctor can disclose a patient’s information to a research team without his permission.	0.24	0.42	43 (23.9)	137 (76.1)
14. A doctor can disclose an adult patient’s information to a specific family member (father, husband, wife) without his permission.	0.26	0.44	47 (26.1)	133 (73.9)
15. A doctor can disclose a patient’s information to the judicial department only with his permission.	0.88	0.32	159 (88.3)	21 (11.7)
16. A doctor can disclose a patient’s information in case of communicable diseases.	0.76	0.42	137 (76.1)	43 (23.9)
17. Visitors have the right to know about the patient’s condition.	0.38	0.48	68 (37.8)	112 (62.2)
18. Procedures or interventions should be briefly discussed with the patient.	0.94	0.24	169 (93.9)	11 (6.1)
19. A consent form is required for both routine and emergent lifesaving procedures.	0.94	0.23	170 (94.4)	10 (5.6)
20. Written consent is required in all procedures even if verbal consent was acquired.	0.96	0.20	172 (95.6)	8 (4.4)
21. Consent must be written in a language understandable by the patient.	0.97	0.18	174 (96.7)	6 (3.3)
22. The patient should be provided with one consent for different interventions like surgery, anesthesia, and radiology.	0.88	0.32	158 (87.8)	22 (12.2)
23. The patient should be aware of both common and rare complications.	0.92	0.26	166 (92.2)	14 (7.8)
24. Treatment procedure should be done even if refused by the patient.	0.65	0.47	117 (65)	63 (35)
25. Doctors are entitled to withhold any procedures related to a patient’s condition if the patient refuses their choice of treatment.	0.84	0.36	152 (84.4)	28 (15.6)
26. To get the patient participation in research, he must be provided with clear and comprehensive information.	0.91	0.28	164 (91.1)	16 (8.9)
27. Patients in governmental hospitals do not have the right to refuse participation in any research done by the hospital.	0.36	0.48	64 (35.6)	116 (64.4)
28. The patient does not have the right to quit after agreeing to participate in research.	0.29	0.45	52 (28.9)	128 (71.1)
29. The patient has the right to know in advance about his treatment cost and insurance coverage.	0.86	0.35	154 (85.6)	26 (14.4)
30. The patient does not need to know about his treatment cost if he was covered by insurance.	0.56	0.49	101 (56.1)	79 (43.9)
31. The patient has the right to request a medical report at any time.	0.87	0.34	156 (86.7)	24 (13.3)
32. The patient has the right to choose the statements to be written in the medical report.	0.82	0.38	148 (82.2)	32 (17.8)
33. Patients have the right to complain to the administration.	0.92	0.26	166 (92.2)	14 (7.80)
34. The medical team should report any violence against children to the concerned authority.	0.96	0.20	172 (95.6)	8 (4.40)
Total score	26.11 ± 2.32			

Most of the nursing students agreed about the patients’ rights of being notified about the diagnosis and all treatments (99.4%), having written consent in an understandable language (96.7%), contacting the treating physician (96.1%), the possibility of obtaining a second opinion within the same hospital or another (96.1%), requiring written consent in all procedures, and reporting any violence against children by the medical team to the concerned authority (95.6%). Unexpectedly, less than half of the participants agreed that patients are not required to be treated with courtesy and respect during times of emergency (47.8%).

On the other hand, nursing students showed inadequate knowledge of the following items in descending orders: visitors have the right to know about the patient’s condition (37.8%), a patient in governmental hospitals does not have the right to refuse participation in any research done by the hospital (35.6%), a patient does not have the right to quit after agreeing to participate in research (28.9%), a doctor can disclose adult patient’s information to a specific family member without the patient’s permission (26.1%), and a doctor can disclose patient’s information to a research team without the patient’s permission (23.9%).

Relationship between age, academic level, scale total score, and knowledge level

As shown in Table 4, age was positively and significantly correlated with academic level (r = 0.522, p < 0.01). Moreover, the participant’s academic level was positively and significantly correlated with the scale total score (r = 0.418, p < 0.01) and participants with adequate knowledge (r = 0.388, p < 0.01), so the participants who have adequate knowledge are in the higher academic level. On the other hand, the participant’s academic level was negatively and significantly correlated with participants with inadequate knowledge (r = -0.388, p < 0.01), so the participants who have inadequate knowledge are at a lower academic level.

**Table 4:** Relationship between age, academic level, scale total score, and knowledge level Note: *p ≤ 0.05, **p ≤ 0.01

Variable	1	2	3	4	5	6
Age	-					
Academic level	0.522**	-				
Total score	0.076	0.418**	-			
Cutoff point	0.076	0.418**	1.00**	-		
Participants with inadequate knowledge	0.025	-0.388**	-0.732**	-0.732**	-	
Participants with adequate knowledge	-0.025	0.388**	0.732**	0.732**	-1.00**	-

In addition, the scale total score was negatively and significantly correlated with participants with inadequate knowledge (r = -0.732, p < 0.01), so the participants with inadequate knowledge have a lower scale total score. In contrast, the scale total score was positively and significantly correlated with participants with adequate knowledge (r = 0.732, p < 0.01), so the participants with adequate knowledge have a higher scale total score.

Reflective experience: factors affecting the ethical knowledge and awareness of the Bill of Rights from students’ perspectives

The number of nursing students who answered the open-ended questions about different factors that shape students’ ethical knowledge and awareness of the Bill of Rights was 100 (55.5%) out of the total sample (N = 180). Students reported many barriers and negative factors that affect their knowledge, such as lack of staff knowledge and compliance with the Bill of Rights, staff shortage, heavy workload (100%), language barriers and lack of patient knowledge (50%), and non-supportive learning and management and hospital culture (41%). All respondents commented that college courses and lectures, university workshops, an ethical climate in the learning environment with Bills of Rights and bylaws, and the presence of an ethical committee and an institutional review board are the main positive factors that influence their ethical knowledge and commitment.

Four students mentioned that experiencing situations that included ethical dilemmas played a role in the perceived importance of the patients’ bill of rights. All students reported that knowing the patients’ bill of rights is very important during their academic study, and they strongly agree that ethics education is important in undergraduate education. Students reported that classroom and clinical training (100%), clinical cases and scenarios (75%), expert ethics educators (33%), and participation in interprofessional education (IPE) sessions with different medical students (30%) are the learning experiences and sources of information that affected their ethics and knowledge of patients’ bill of rights. Some of the students’ reflective statements are included in Table 5, as well as their responses to open-ended questions.

**Table 5:** Factors affecting the ethical knowledge and awareness of the Bill of Rights from students’ perspectives (N = 100) *Multiple responses by one participant

Open-ended questions	Number	%
What are the barriers or negative factors that influence nursing students’ knowledge of patients’ bills of rights?* (N = 100)		
Lack of knowledge and compliance about the bill among staff themselves	100	100
Staff shortage and heavy workload lead them to be more task-oriented	100	100
Language and interpersonal communication barriers between Saudi patients and non-Arabic nurses	50	50
Lack of patients’ knowledge about their bill of rights	50	50
Non-supportive learning and hospital management culture toward the Bill of Rights	41	41
What are the facilitating factors that influence nursing students’ knowledge of patients’ bills of rights?*(N = 100)		
College courses and lectures on the ethical and moral rights of the patients in each specialty	100	100
Different workshops done in the university about ethical considerations in teaching and research	100	100
Cultivation of an ethical climate in the learning environment through students’ bills of rights and bylaws, which help build the idea of having the Bill of Rights everywhere and for every person	100	100
The presence of an ethical committee and an institutional review board	100	100
A situation or a story that shaped your perception of the importance of the patients’ bill of rights (N = 4)		
Having a personal experience and problem with a family member’s right to access care on time	-	-
The situation with the non-protection of patient privacy by the intern nurse	-	-
A patient complains about a physician who neglected to inform him of the detailed treatment plan	-	-
A nurse discloses private information about the patient in the nursing station and the nurse manager’s action	-	-
How do you rate the importance of knowing the patients’ bill of rights during your academic studies (from 1 to 5 (from very important to not important)) and why?		
Very important	100	100
Sample student reflective statement: “Knowing the Bill of Rights helps in maintaining an ethical hospital climate that respects patients and helps nurses and other healthcare providers provide quality care according to patient needs; it also helps protect nurses against legal suits.”		
To what extent do you agree that ethics education is important in undergraduate education, and why?		
Strongly agree	100	100
Sample student reflective statement: “Ethics are very important in undergraduate education. It helps students know the laws that govern nursing practice, the code of ethics, and common ethical principles. It also helps in identifying the Islamic and religious perspectives and sources of support for patient rights. Ethical education is a significant feature of nursing as a caring profession.”		
Which learning experience or sources of information affected your ethics knowledge and knowledge of patients’ bill of rights?*		
Classroom and clinical training	100	100
Clinical cases and scenarios we discussed in the clinical training	75	75
Having expert ethics educators	33	33
Participation in interprofessional education sessions with different medical students	30	30
Sample student reflective statement: “Both classroom learning and clinical training are important because we get the theoretical information from classroom teaching, and we are gaining real-life experience and dealing with patient cases from the clinical training in the hospital. Interprofessional education sessions with different medical students and clinical-based scenarios are effective strategies to improve ethics education and our knowledge.”		

## Discussion

Limited research studies investigated the knowledge of the patients’ bill of rights among nursing students, so we tried our best to discuss our findings in light of available research in the medical field. Our discussion starts with the self-reported levels of knowledge of patients’ bill of rights, followed by factors influencing this knowledge.

According to the Bill of Rights questionnaire, the level of knowledge was categorized as adequate if the score was ≥75 (score of 26-34) and inadequate knowledge if the score was <75 (score of 25). The current study revealed that the total knowledge score about patients’ bill of rights ranged from 19 to 34, with an overall mean of 26.11 (76.8% of the total score), indicating that participants had adequate knowledge of most of the items of the patients’ bill of rights, where the score of knowledge was ≥75%. Comparable to the findings from Al Anazi et al. [22], who surveyed 205 medical students, they indicated that the total knowledge score of medical students about the patients’ bill of rights ranged between 0 and 32 with a mean of 24.6 (72.4% of the total score), which reflects inadequate knowledge about the patients’ bill of rights as it is lower than 75% of the total score. Our result could be attributed to the effects of academic study and courses. Students were introduced to the specialized course about nursing ethics and patient safety at their fifth academic level, and they were aware, to some extent, of ethical principles, human rights, and patient rights. This perspective could be supported by the findings of Alnajjar and Abou Hashish [12], which revealed that nursing students showed moral sensitivity to patient-oriented care and professional responsibility and linked their findings to the influence of nursing ethics education provided to students by their fourth and fifth academic levels. A similar finding was also reported by Yeom et al. [23], who stated that nursing ethics education had a substantial influence on the moral sensitivity toward patient-oriented care among nursing students.

On the other hand, the current study indicated that about one-third of nursing students (34.5%) have an inadequate level of knowledge of the Bill of Rights. This result is similar to some extent to that of Kupcewicz et al. [3], who included undergraduate nursing students from three European countries: Slovakia, Poland, and Spain. They indicated that 35.9% of nursing students from Slovakia, 26.5% from Poland, and 14.9% from Spain had a low level of knowledge of patients’ rights. In contrast, Kiong et al. [4] indicated that 48.22% of medical students had inadequate knowledge about patients’ rights. Compared with our study in the Saudi context, El-Sobkey et al. [6] found that 92% of the students of applied medical sciences surveyed did not know any of the items on the Bill of Rights. Moreover, Dabbagh et al. [24] studied knowledge about the patients’ bill of rights among a sample of visitors; 85% of them were students of medical sciences, which showed students’ limited knowledge regarding the existence of the Saudi Patients’ Bill of Rights and its contents. Other studies conducted among patients and healthcare providers in Saudi Arabia showed inadequate knowledge about the patient’s bill of rights among primary healthcare clinics [10] and recipients of health services in Saudi Arabia [18]. The differences among studies might be related to the differences in the level of education, experience, and responsibility between students and providers or recipients of care. In this regard, Dabbagh et al. [24] highlighted the necessity of raising the awareness of both providers and patients and directing efforts toward providing better methods for educating them and involving patients in shared decision-making for improving the quality of healthcare services.

Factors affecting students’ ethical knowledge and awareness of the Bill of Rights

Our study indicates that age and academic level are positively related to students’ ethical knowledge. It means that the higher the students’ academic level, the more knowledge they have about the patients’ bill of rights. In other words, nursing students at level 12 have more knowledge than those at level 11. One possible explanation for this correlation is that having a higher academic level always entails having more opportunities for clinical training and observing nurses as they interact with patients in a real-world setting. Experience and familiarity with patient cases provide more knowledge about patients’ rights. Similarly, Alnajjar and Abou Hashish [12] found that academic level positively affects students’ ethical awareness, and they reported that senior students displayed a higher level of awareness than younger students. They related their findings to academic experience, knowledge of college life, and clinical training. Moreover, the same result was indicated by Al Anazi et al. [22]. However, it is not clear whether the positive correlation between age, academic level, and students’ ethical knowledge is a result of increased clinical training and observing nurses in real-world settings or whether other factors might be responsible for this relationship. Hence, a future study is recommended to establish the causal effect of age and academic level on ethical knowledge.

In addition to nurses’ age and academic level, students reported many factors that shape their ethical knowledge and awareness of the Bill of Rights from their perspectives. All students who responded to open-ended questions commented that college courses and lectures, university workshops, an ethical climate in the learning environment with bylaws, and the presence of an ethical committee and an institutional review board are the main positive factors that influence their ethical knowledge and commitment. Students also reported that knowing the patient’s bill of rights is very important during their academic studies, and they strongly agree that ethics education is important in undergraduate education. Four students mentioned that experiencing situations that included ethical dilemmas played a role in the perceived importance of the patient’s bill of rights. This result could be supported by Johari et al. [25], who emphasized the importance of an ethical climate and argued that any organization’s professional climate is supported by awareness, training, and a code of conduct. They indicated that conducting forums for discussion of ethical issues, policies, and institutional codes would improve and enhance the ethical climate. Other studies also emphasized the role of nurse educators in fostering a learning environment conducive to ethical awareness and professional values [11,12]. Recently, Hussein and Abou Hashish [11] stated that ethical concepts such as ethical ideologies, professional values and sensitivity, judgment, and developing nursing accountability in dealing with ethical challenges that they may encounter in their professional career require greater emphasis and application in nursing curricula. Likewise, Abedian et al. [2] illustrated the positive effect of education and training interventions in improving awareness and practice by nurses on the patient’s bill of rights.

On the other hand, students reported many barriers and negative factors that affect their knowledge, such as a lack of staff and patients’ knowledge and compliance about the Bill of Rights, a staff shortage, a heavy workload, language barriers, non-supportive learning and management, and hospital culture. Alghanim [21] listed comparable barriers that might prevent the PBR’s implementation in Saudi Arabia, such as a lack of knowledge about the bill among patients and staff, a shortage of staff, a heavy workload, and inadequate resources. In addition, he reported time constraints, an improper staff/patient ratio, and an unsafe environment. In a related study, Alsayed et al. [26] also mentioned that staff shortages, increased workload, and demands on nurses affect their performance and well-being, as well as patient safety and rights. Also, language and interpersonal communication barriers were reported as negative factors for maintaining the Bill of Rights. This result could be attributed to the nursing workforce in Saudi Arabia, where nursing is dominated by expatriate non-Saudi nurses from various countries, which creates challenges linked to cultural, ethical, and religious differences and communication barriers. Similarly, Alshammari et al. [27] found that language, religion, and cultural differences among nurses who provide health services in Saudi Arabia can directly influence nurse-patient communication and relationships. However, the Saudization program and the provision of soft skills and communication training programs would be helpful strategies [26,27].

Strengths, limitations, and future research

The current study contributes to Saudi nursing, especially among nursing students, and provides fruitful insights into the knowledge and awareness level of the PBR. It also sheds light on the different factors that might influence students’ knowledge, attitude, and skills in the clinical setting in relation to ethics and patient rights, which were not explored or examined in the previous research. However, our study was not without limitations. The sample represented nursing students at a single institution, and their perceptions may not represent the entire Saudi nursing student population, thus reducing the generalizability factor in our study. Therefore, it may be helpful for future researchers to compare the level of knowledge among a larger sample from different colleges. In addition, the college is primarily composed of female students, and gender may impact perception. Besides, the respondents in our study might have given whatever they thought would be an acceptable response to the researchers rather than revealing the whole truth or picking the answers that resonated with them, and this might include bias because of social desirability. Thus, we recommend conducting future observational and longitudinal studies.

Although we included open-ended questions to elicit more information from students’ perspectives about factors influencing their knowledge, we recommend having a qualitative study about students’ lived experiences. As we did not use intervention or regression analysis and did not establish a causal relationship between the identified factors and students’ knowledge level, we recommended future studies to establish a causal relationship. Also, we recommend a study about the perceived importance and knowledge of the patients’ bill of rights from the perspectives of students, patients, and healthcare providers (including nurses) for a more comprehensive view and tailored improvement strategies for implementation. A future qualitative assessment of patients’ attitudes toward practicing their rights and comparing them with nurses’ perspectives is also crucial for empowering patients and improving their capabilities in shared decision-making.

## Conclusions

In conclusion, our findings provide a knowledge base among Saudi nursing students about the Bill of Rights and the many factors that affect their knowledge and practice readiness. Our main results indicated that about two-thirds of nursing students reported adequate knowledge of the patients’ bill of rights. Age, academic level, prior ethical awareness, and ethics education positively affected their perception. However, there is still a gap in their knowledge level, with 34.5% of students having inadequate knowledge about the patients’ bill of rights. Although many years have passed since the publication and enforcement of the Patients’ Bill of Rights at healthcare organizations in Saudi Arabia, knowledge about these rights is still lacking in the community, particularly among the nursing student population.

Students reflected on many factors that affected their perception and knowledge, and they referred to many of these facilitators as recommendations in our study. Integrated classroom-clinical education, interprofessional learning experiences, workshops, a supportive and ethical learning environment, and the presence of an ethical committee seemed to positively impact their knowledge. Therefore, we recommend a continuous effort to foster ethics education with inspiring learning content and innovative instructional material that are vital to improving nursing students’ knowledge and readiness to provide ethical care. Interprofessional education sessions and clinical-based scenarios seem like effective strategies to improve ethics education and awareness.

The implementation of college- and hospital-based education interventions for nursing students and nurses will increase their awareness of and adherence to the patients’ bill of rights. Thus, revising the curriculum for nursing students is recommended, considering relevant and appropriate content in in-service educational programs. Nurse educators and managers should also extensively monitor students’ and nurses’ professional ethics and regulations. In addition to the future studies that are recommended in the limitation section, it is suggested that in future studies, intervention studies based on active learning methods such as role-play and simulation be implemented in the real workplace environment to help increase the depth and extent of education.
